# Association between the physical activity level in the third trimester of pregnancy and the gestational age at birth

**DOI:** 10.1590/1806-9282.20241509

**Published:** 2025-05-02

**Authors:** Tugba Kinay, Sule Atalay Mert, Rahmi Sinan Karadeniz, Yaprak Engin Ustun

**Affiliations:** 1 University of Health Sciences, Etlik Zubeyde Hanim Women's Health Training and Research Hospital, Department of Obstetrics and Gynecology – Ankara, Turkey.; 2 Ankara Etlik City Hospital, Department of Obstetrics and Gynecology – Ankara, Turkey.

**Keywords:** Physical activity, Labor onset, Prolonged pregnancy, Birth

## Abstract

**OBJECTIVE:**

The aim of this study was to evaluate the association between the physical activity level in the third trimester and the time of labor onset.

**METHODS:**

Two hundred and sixty women with low-risk pregnancies, who gave birth at 37 weeks of gestation or beyond, and completed the Pregnancy Physical Activity Questionnaire were included in this prospective, cross-sectional study. According to the gestational age at delivery, the study population was divided into case (≥41 weeks) and control (37–40^6/7^ weeks) groups. The clinical characteristics and the physical activity levels of the two groups were compared. The physical activity levels of the participants were also compared according to the delivery route.

**RESULTS:**

The nulliparity rate (54.3 vs. 21.7%), the median gestational weight gain (10.5 [2–30] vs. 10 [2–25] kg), and the cesarean delivery rate (27.7 vs. 6.6%) were higher in the case group than the control group (p<0.05). While the median level of sedentary activity was higher, the median moderate-intensity activity level and the median household/caregiving activity level were lower in the case group than the control group (p<0.05). The level of sedentary activity was also higher in women who gave birth by a cesarean section than vaginally (p<0.05).

**CONCLUSION:**

Physical activity in the third trimester was associated with the time of labor onset. Decreased moderate-intensity and household/caregiving activity levels and an increased level of sedentary activity in the last trimester of pregnancy were found in women who gave birth at ≥41 weeks of gestation. A decreased level of sedentary activity was observed in women who gave birth vaginally.

## INTRODUCTION

Physical activity is the bodily movement caused by the contraction of skeletal muscles^1^. Physical activity during pregnancy has many benefits, such as preventing excessive gestational weight gain, reducing the risk of gestational diabetes and hypertension, decreasing cesarean section rates, and improving maternal and neonatal outcomes^1-5^.

Labor is a physiological process that leads the fetus to be expelled from the uterus. The timing of labor is controlled by complex interactions between the fetus, mother, and placenta. Various paracrine/autocrine events and fetal hormonal changes trigger the parturition cascade that is responsible for the timely onset of labor^6,7^. Early or late initiation of the labor process leads to preterm birth or postterm pregnancies that could become the cause of serious maternal and perinatal adverse outcomes^8,9^. However, the factors influencing the physiological processes that regulate the labor process and onset time have not been fully revealed yet. The effects of exercise on endocrine hormones are known^10,11^. The hormonal changes caused by physical activity could have an effect on the labor onset in pregnant women.

There are limited reports investigating the association between physical activity and the time of labor onset in term pregnancies. Conflicting results have been reported in these studies. Owe et al.^12^ found that exercising during pregnancy increases the risk of postterm birth. In other studies, it has been reported that there is no relationship between regular exercise during pregnancy and the delivery time^2,13,14^. In most of these studies, physical activity levels were evaluated by using questionnaires including only queries about leisure-time exercises, such as running, jogging, bicycling, swimming, and aerobics^12-14^. However, it is known that many women reduce their leisure-time physical activity during pregnancy^15^, and the most energy-consuming activities are the household and caregiving activities in pregnant women^16^. For this purpose, we aimed to evaluate the association between the physical activity level and the time of labor onset by using a questionnaire including household and caregiving activities, i.e., Pregnancy Physical Activity Questionnaire (PPAQ)^17^. We also aimed to evaluate the association between the physical activity level in the third trimester and the delivery route as a secondary outcome.

## METHODS

This prospective, cross-sectional study was performed in the obstetrics unit of a tertiary care center between May 2022 and April 2023. The study protocol was approved by the local ethical committee (2022/57) and complied with the Helsinki Declaration. A signed informed consent form was obtained from all participants.

Women with low-risk pregnancies, who gave birth at 37 weeks of gestation or beyond, and completed the PPAQ were included in the study. Low-risk pregnancy was defined as a singleton pregnancy older than 18 years of age and without systemic diseases, malpresentation, placenta accreta spectrum, placenta previa, intrauterine growth restriction, polyhydramnios, preterm premature rupture of membrane, and preterm delivery. Women with high-risk pregnancies including the age under 18 years; having multiple pregnancies, polyhydramnios, intrauterine growth restriction, preeclampsia, hypertension, diabetes mellitus, systemic diseases, orthopedic and neurological diseases, malpresentation, placenta previa, and a history of previous uterine surgery or cesarean delivery; who gave birth at <37 weeks of gestation; who did not volunteer to participate in the research; and who do not speak Turkish language were excluded from the study.

The demographic and clinical characteristics of the women were obtained from the medical records. The gestational age of the participants was evaluated by using the last menstrual period and ultrasonography findings that were performed before 20 weeks of gestation. The type, duration, and frequency of the physical activities performed by the women in the third trimester of pregnancy were ascertained using a PPAQ^17^ that had been validated for the Turkish language previously^18^. The questionnaire instructions and the Compendium-based metabolic equivalent of task (MET) values^19^ were used to estimate the intensity of activities. The activities were classified according to their intensity: sedentary activity with a MET value of <1.5, light-intensity activity with a MET value of 1.5–3.0, moderate-intensity activity with a MET value of 3.0–6.0, and vigorous activity with a MET value of >6. The weekly energy expenditure (MET-h.wk^-1^) was computed by multiplying the time spent on each activity by its intensity. The average numbers of MET-h.wk^-1^ in each intensity level (sedentary, light, moderate, vigorous, and total) and in each activity type (household/caregiving, occupational, and sports/exercise) were calculated.

According to the gestational age at delivery, the study population was divided into case and control groups: the case group included women who gave birth at ≥41 weeks of gestation and the control group included women who gave birth at 37–40^6/7^ weeks of gestation. The physical activity levels, the average number of the MET-h.wk^-1^ expended, were compared between the two groups. The physical activity levels were also evaluated according to the delivery route.

A power analysis was carried out to determine the sample size. When the correlation coefficient between the gestational age and the level of physical activity is predicted to be r=-0.176, the sample size required for 80% power with a 5% type I error level is calculated as at least 251 people^20^. The statistical analysis was performed using IBM SPSS Statistics software version 22.0 (SPSS Inc., Chicago, IL, USA). Descriptive statistics were presented as median (min–max) values for continuous variables and the number and percentage for categorical variables. The normality of continuous variables was evaluated by using Kolmogorov-Smirnov and Shapiro-Wilk tests. Mann-Whitney U test was used for the analysis of non-parametric data. The differences between the case and control groups in terms of categorical variables were examined using Pearson's chi-square test. The ability of physical activity levels to predict the gestational age at delivery was evaluated using receiver operating characteristic (ROC) analysis. Optimal cutoff points were estimated for the MET values of sedentary, moderate-intensity, and household/caregiving activities. p-values less than 0.05 were accepted as statistically significant. A multiple logistic regression analysis was carried out to determine the independent risk factors for the birth at ≥41 weeks of gestation.

## RESULTS

During the study period, the medical records of 278 women who gave birth at ≥37 weeks of gestation and met the inclusion criteria were evaluated. Eighteen women were excluded from the study due to incomplete questionnaire and data. Hence, from the remaining 260 women, 94 who gave birth at ≥41 weeks of gestation and 166 who gave birth at 37–40^6/7^ weeks of gestation were included in the study.

The demographic and clinical characteristics of the case and control groups are shown in Table 1. The median age and body mass index (BMI) of the two groups were similar (p>0.005). The nulliparity rate (54.3 vs. 21.7%, p<0.001) and the median gestational weight gain (10.50 [2.0–30.0] vs. 10.0 [2.0–25.0] kg, p=0.005) were higher in the case group than the control group. The cesarean delivery rate was higher in the case group than the control group (27.7 vs. 6.6%, p<0.001).

**Table 1:** Demographic and clinical characteristics of the case and control groups.

Characteristics		Case group (≥41 weeks) n=94	Control group (37–40^6/7^ weeks) n=166	p-value
Age, years		26 (18–38)	26.5 (18–43)	0.123
BMI, kg/m^2^		28.89 (21.45–41.52)	28.44 (19.47–40.37)	0.275
Parity		0 (0–5)	1 (0–6)	<0.001
Nulliparity		51 (54.3%)	36 (21.7%)	<0.001
Gestational weight gain, kg		10.5 (2–30)	10 (2–25)	0.005
Working		7 (7.5%)	7 (4.3%)	0.285
Smoking		6 (6.4%)	15 (9.6%)	0.380
Birth interval for multiparous women, years		5 (1–10)	3 (1–14)	0.025
Previous child birthweight, g		3,200 (1,300–4,100)	3,365 (1,300–4,800)	0.383
Labor induction		50 (53.2%)	–	
Labor induction method				
	Balloon	25 (50.0%)	–	
	Oxytocin	20 (40.0%)	–	
	Misoprostol	2 (4.0%)	–	
	Dinoprostone	3 (6.0%)	–	
Delivery route				<0.001
	Vaginal	68 (72.3%)	155 (94.5%)	
	Cesarean section	26 (27.7%)	11 (6.6%)	
Newborn birthweight, g		3,350 (2,460–4,430)	3,230 (2,310–4,250)	0.073

BMI: body mass index.

As shown in Table 2, the physical activity levels of the case and control groups were different. While the median level of sedentary activity was higher, the median moderate-intensity activity level and the median household/caregiving activity level were lower in the case group than the control group (p<0.05).

**Table 2:** Physical activity levels of the case and control groups.

Physical activity level	Case group (≥41 weeks) (MET-h.wk^-1^)	Control group (37–40^6/7^ weeks) (MET-h.wk^-1^)	p-value
Total activity	169.39 (4.38–677.30)	173.78 (4.38–455.00)	0.953
Sedentary activity	49.70 (1.93–163.28)	25.11 (1.75–106.93)	<0.001
Light-intensity activity	94.06 (4.38–242.55)	86.28 (4.38–224.35)	0.807
Moderate-intensity activity	54.25 (0.80–299.50)	72.95 (2.40–266.00)	0.037
Vigorous-intensity activity	5.25 (1.63–37.25)	1.75 (1.63–16.25)	0.262
Household/caregiving activity	99.93 (4.38–405.65)	133.79 (4.38–45.00)	0.011
Occupational activity	75.25 (3.85–119.18)	100.01 (67.20–194.08)	0.286
Sports/exercise	7.30 (0.80–102.50)	4.80 (0.80–29.78)	0.013

According to the ROC analysis, the optimal sedentary activity cutoff level for predicting birth risk at ≥41 weeks of gestation was 35.70 MET-h.wk^-1^ (area under the curve [AUC], 0.712; 95%CI 0.637–0.787; sensitivity, 67.1%; specificity, 60.1%; p<0.001). A 64.02 MET-h.wk^-1^ cut-off level of moderate-intensity activity was found for predicting birth at < 41 weeks of gestation (AUC, 0.584; 95%CI, 0.505-–0.662; sensitivity, 58.6%; specificity, 55.7%; p = =0.037) and a 117.86 MET-h.wk^-1^ cut-off level of household/caregiving activity was found for predicting birth at < 41 weeks of gestation (AUC, 0.597; 95%CI, 0.523-–0.670; sensitivity, 60.4%; specificity, 57.8%; p = =0.011).

As shown in Table 3, the physical activity level in the third trimester was also associated with the delivery route. While the median level of sedentary activity was lower, the median occupational activity level was higher in the women who gave birth vaginally than by a cesarean section (p<0.005). The Association between demographic and clinical variables (parity, gestational weight gain, birth interval, level of sedentary activity, moderate-intensity activity level, and household/caregiving activity level) and delivery at ≥41 weeks of gestation was modeled by a logistic regression analysis. According to the multiple logistic regression analysis results, the level of sedentary activity (odds ratio [OR] 1.03; 95% CI 1.02–1.05) was an independent risk factor for the birth at ≥41 weeks of gestation.

**Table 3:** Physical activity levels of the women who gave birth vaginally and by a cesarean section.

Physical activity level	Vaginal delivery (n=223) (MET-h.wk^-1^)	Cesarean delivery (n=37) (MET-h.wk^-1^)	p-value
Total activity	175.30 (23.28–677.30)	177.72 (82.78–359.92)	0.545
Sedentary activity	32.99 (1.75–163.28)	50.84 (5.08–96.08)	0.011
Light-intensity activity	103.86 (5.43–242.55)	83.21 (36.75–224.35)	0.874
Moderate-intensity activity	74.80 (0.80–299.50)	63.73 (10.55–115.30)	0.128
Vigorous-intensity activity	4.88 (1.63–16.25)	5.25 (1.63–37.25)	0.932
Household/caregiving activity	108.90 (13.52–405.65)	75.60 (58.80–123.20)	0.061
Occupational activity	108.24 (67.20–194.08)	29.93 (3.85–56.00)	0.040
Sports/exercise	8.00 (5.18–29.78)	41.18 (3.55–4.80)	0.094

## DISCUSSION

The study results showed that while the level of sedentary activity was higher, the moderate-intensity activity level and the household/caregiving activity level were lower in women who gave birth at ≥41 weeks of gestation than at 37–40^6/7^ weeks of gestation. The level of sedentary activity was lower in women who gave birth vaginally than by a cesarean section.

It is known that continued physical activity during pregnancy has many benefits for both the mother and the infant. Previous studies have reported a lower risk of preeclampsia, gestational diabetes mellitus, and cesarean delivery in physically active pregnant women^3-5^. Studies investigating the association between the physical activity level and the time of labor onset have shown that physical activity during pregnancy reduces the risk of preterm births^12^. There is a limited number of reports on the effect of physical activity during pregnancy on postterm pregnancies, and these studies have conflicting results^2,12,21^. In the series of Haakstad et al.^2^ and Evenson et al.^21^, no association was found between the postterm birth (≥42 weeks of gestation) and the physical activity level during pregnancy. On the other hand, Owe et al.^12^ reported that women exercising 3–5 times per week in the gestational week of 17 were more likely to have a postterm birth. Differently, we investigated the relationship between the physical activity level in the third trimester and the time of labor onset. We found that the increased physical activity level in the third trimester reduced the birth risk at ≥41 weeks of gestation.

In most of the studies reporting the relationship between the physical activity level and the time of labor onset, the physical activity level of the cases had been evaluated by using questionnaires including queries about only leisure-time exercises^12-14^. However, studies have shown that most women do not continue their pre-pregnancy exercises during pregnancy^15,22^. In a study on 1,737 women, Fell et al.^22^ reported the impact of pregnancy on the physical activity level, including household and caregiving activities, active living, sports, and exercise activities. They showed that the largest decrease occurred in sports and exercise activities during the first 20 weeks of pregnancy compared with the year prior to pregnancy. Therefore, physical activity questionnaires that do not include household and caregiving activities could lead to inaccurate determination of the physical activity level in pregnant women and misunderstanding about the correlation between the physical activity level and the outcomes. In our study, we used the PPAQ that includes queries about the household and caregiving activities and has good validity and reliability characteristics^23^. Unlike the research assessing only leisure-time exercises for the determination of the physical activity level during pregnancy, we evaluated the impact of different physical activity intensities and types on the time of labor onset. In our series, the moderate-intensity activity level and the household/caregiving activity level were lower and the level of sedentary activity was higher in women who gave birth at ≥41 weeks of gestation than at 37–40^6/7^ weeks of gestation.

It has been previously reported that physical activity during pregnancy positively affects the labor and delivery outcomes. Watkins et al.^24^ reported the shorter active labor duration and the reduced risk of prolonged first stage of labor in women with a high physical activity level in the third trimester of pregnancy. In a recent meta-analysis, decreased cesarean section rates were reported in physically active pregnant women^5^. Similarly, we found an association between the delivery route and the physical activity level in the third trimester. The median level of sedentary activity was lower and the median occupational activity level was higher in women who gave birth vaginally than by a cesarean section in the study cohort. According to the study results, increased physical activity in pregnant women during the last trimester could lead to a timely onset of labor and a decrease in the need for cesarean delivery, thus reducing the risk of adverse maternal and neonatal outcomes.

The present study is one of the limited studies in the literature reporting the association between the physical activity level and the time of labor onset. The homogeneous study population is one of the strengths of the study. Only low-risk term pregnancies were included in this study. Pregnant women with diseases that could affect the labor onset were excluded. The selected study population of low-risk term pregnancies may have influenced the study results and introduced potential bias. Studies with a larger sample size including all low- and high-risk pregnancies are required to confirm our results. Another strength of the study was the measurement method used for the assessment of physical activity. The validated PPAQ is a simple-to-use, cost-effective method to evaluate physical activity and it was designed specifically for pregnant women. Nevertheless, the PPAQ was also the major limitation of the study because this self-reported questionnaire reflected the perceptions of those attitudes rather than the actual attitudes on the subject and was prone to measurement error and bias.

## CONCLUSION

The study results showed that the physical activity level in the third trimester was associated with the gestational age at birth. The increased moderate-intensity and household/caregiving activities and the decreased sedentary activities in the last trimester of pregnancy could prevent the delay in the onset of labor that could lead to severe maternal and perinatal adverse outcomes. The physical activity level in the third trimester was also associated with the delivery route. Women who gave birth vaginally had lower levels of sedentary activity and higher levels of occupational activity than women who underwent a cesarean section.
